# A Question of Frame: The Role of the Bone Marrow Stromal Niche in Myeloid Malignancies

**DOI:** 10.1097/HS9.0000000000000896

**Published:** 2023-05-23

**Authors:** Chiara Tomasoni, Alice Pievani, Benedetta Rambaldi, Andrea Biondi, Marta Serafini

**Affiliations:** 1 Tettamanti Center, Fondazione IRCCS San Gerardo dei Tintori, Monza, Italy; 2 Hematology and Bone Marrow Transplant Unit, ASST Papa Giovanni XXIII, Bergamo, Italy; 3 Pediatrics, Fondazione IRCCS San Gerardo dei Tintori, Monza, Italy; 4 Department of Medicine and Surgery, University of Milano-Bicocca, Monza, Italy

## Abstract

Until a few years ago, the onset of acute myeloid leukemia (AML) was entirely ascribed to genetic lesions in hematopoietic stem cells. These mutations generate leukemic stem cells, which are known to be the main ones responsible for chemoresistance and relapse. However, in the last years, increasing evidence demonstrated that dynamic interplay between leukemic cells and bone marrow (BM) niche is of paramount relevance in the pathogenesis of myeloid malignancies, including AML. Specifically, BM stromal niche components, such as mesenchymal stromal cells (MSCs) and their osteoblastic cell derivatives, play a key role not only in supporting normal hematopoiesis but also in the manifestation and progression of myeloid malignancies. Here, we reviewed recent clinical and experimental findings about how genetic and functional alterations in MSCs and osteolineage progeny can contribute to leukemogenesis and how leukemic cells in turn generate a corrupted niche able to support myeloid neoplasms. Moreover, we discussed how the newest single-cell technologies may help dissect the interactions between BM stromal cells and malignant hematopoiesis. The deep comprehension of the tangled relationship between stroma and AML blasts and their modulation during disease progression may have a valuable impact on the development of new microenvironment-directed therapeutic strategies, potentially useful for a wide cohort of patients.

## INTRODUCTION

Acute myeloid leukemia (AML) is a hematopoietic malignancy originating from hematopoietic stem cells (HSCs) and progenitor cells. The annual incidence is around 2.4 cases per 100,000 individuals and it increases progressively with age, to a peak of 12.6 per 100,000 in adults of 65 years of age or older.^1^ A proportion of AML cases does not present as a de novo disease but represents the clinical evolution of clonal HSC disorders such as myelodysplastic syndromes (MDS) or myeloproliferative neoplasms (MPN). In recent years, there have been major advances in our understanding of AML, including new knowledge about the molecular pathogenesis, leading to an update of the disease classification,^2,3^ technological progress in genomic diagnostics and assessment of minimal residual disease,^4,5^ and the successful development of new therapeutic agents.^6^ Disease-specific chemotherapy regimens and consolidation with hematopoietic cell transplant (HCT) have enhanced AML outcomes, reaching an overall survival of ~60% at 5 years across different studies,^7,8^ and chemo-free regimens have opened new possibilities for elderly patients not eligible for chemotherapy treatment.^9^ Despite that, AML patients still face a risk of treatment-resistance and relapse that severely affects the outcome. Furthermore, the application of new targeted therapies into clinical practice remains challenging due to the high complexity of this disease, including multiple driving mutations and the coexistence of several competing tumorigenic clones. These persistent difficulties necessitate the identification of innovative therapeutic approaches that are effective for a larger cohort of AML patients.

The main cause of relapse in AML patients is the presence of chemoresistant leukemic stem cells (LSCs) nested in the bone marrow (BM) niche.^10^ LSCs are characterized by quiescence and self-renewal capability, which make them able to induce AML onset when transplanted in secondary and preferentially tertiary recipient mice and to sustain cell survival in optimized ex vivo co-culture systems.^11^ LSCs arise from the accumulation of somatic DNA mutations in HSCs in genes involved in quiescence, cell cycle, and cell proliferation (*FLT3, RAS, P53, c-KIT*, and *STAT3*), self-renewal potential, differentiation capability (*NPM1* and *CEBPA*), and epigenetic regulation such as DNA-methylation-related genes (*DNMT3A*, *TET2*, and *IDH-1*/*2*).^12^ Advances in cancer genomics have revealed that most AML genomes display a small number of mutations, which are acquired in a stepwise manner (named the two-hit theory).^13^ LSC therapeutic targeting and elimination are mandatory to improve the dismal AML outcome. However, LSCs not only share surface markers with HSCs and AML blasts, but also present a large heterogeneity within and among patients, and thus the identification of therapeutic LSC-specific targets is challenging.^14^

The BM niche is a complex network of molecular and cellular entities composed of mesenchymal stromal cells (MSCs), endothelial cells, osteoblastic cells, nerves from the sympathetic nervous system, and accessory cells (eg, macrophages, megakaryocytes, and T cells). It plays a role not only in BM homeostasis but also in hematological malignancies, such as myeloid diseases.^15^ Indeed, malignant cells reside within the niche and extensively communicate with its components. These interactions contribute to the initiation of malignancy, support of disease progression, resistance to chemotherapy, and loss of normal hematopoiesis.

This review describes the latest findings regarding the interplay between myeloid malignancies, such as AML, MDS and MPN, and the BM stromal niche, focusing on clinical and experimental aspects that support the pivotal role of the MSCs and their osteoblastic progeny in supporting disease initiation and progression. Unraveling the molecular basis of these interactions, by using new sophisticated imaging and sequencing technologies, is a prerequisite for identifying new targetable cell-extrinsic factors in the BM niche that, in addition to the classical targeting of leukemic cell-intrinsic mechanisms, can improve the therapeutic benefit for these patients.

## ALTERATIONS OF BM NICHE IN MYELOID MALIGNANCIES: CLINICAL EVIDENCE

The BM niche can become altered during hematopoietic malignancies, as it has been demonstrated in multiple murine models of leukemia and has been very well-described in patients with multiple myeloma.^16,17^ Regarding myeloid diseases, the most striking evidence of BM niche alteration in patients is the BM fibrosis that occurs during the development of MPN, especially in myelofibrosis (MF). Although intrinsic MSC alterations have been observed in MF patients,^18,19^ pathological HSCs play a pivotal role in subverting the BM niche.^20^ This concept is reinforced by data from patients that recovered after HCT where a complete response is defined by the complete regression of BM fibrosis.^21^

In AML patients, some BM morphological features that correlate with the outcome have been described. In the study of Lu and colleagues^22^, the authors analyzed the size of marrow adipocytes in BM sections from 70 patients with primary AML, observing a correlation between increased amounts of small BM adipocytes and a poorer prognosis. Another study showed that BM biopsies of AML patients present a significant increase of CD271^+^ MSCs, which in turn enhance the production of reticular fibers,^23^ commonly associated with therapy failure and decreased overall survival.^24^ Several studies reported an increased number of vessels and other vascular abnormalities in AML patient-derived BM biopsies, raising the interest in antiangiogenic therapies also in AML.^25^ However, whether increased angiogenesis constitutes a prognostic factor for AML treatment response remains unclear. Furthermore, a decrease of osteocalcin (OCN), a noncollagenous protein strictly correlated with bone turnover, was found in BM and peripheral blood serum of AML patients, especially those with adverse-risk disease.^26^ Notably, a reduced OCN level correlates to inferior overall survival, poor response to chemotherapy, and lower relapse-free survival in AML patients. Moreover, chemotherapeutic treatments can cause a restoration of OCN levels as a consequence of an increase in osteoblast (OB) number in the BM.^23,27^

The BM microenvironment is implicated in drug resistance not only by changing the pharmacokinetics of drugs by the alteration of BM vascular permeability,^28^ but also by specific signaling pathways triggered by the altered BM components, especially MSCs. Indeed, it has been extensively described how MSCs endow AML cells with resistance to several therapeutic agents, including standard chemotherapy and new molecular targeted agents such as Fms-like tyrosine kinase (FLT3) inhibitors (midostaurin, sorafenib, and quizartinib). Co-cultures of AML cells harboring internal tandem duplication of *FLT3 (FLT3-ITD*) together with MSCs protect blasts from FLT3 inhibitors through the alteration of cytokines, chemokines, RAS/MAPK, and ERK signaling pathways.^29^ However, the precise population of cells and molecular mechanisms involved in chemoresistance have only been partially elucidated.

An important demonstration of the role of the BM niche in protecting leukemic cells from chemotherapy comes from the clinical use of the CXCL12/CXCR4 axis inhibitors. Indeed, different groups have tried to boost chemotherapy activity by stripping the LSCs from their niche, upon administration of granulocyte colony-stimulating factor (G-CSF) or plerixafor in combination with antileukemic treatments, with promising results.^30,31^ In particular, Uy and colleagues^31^ demonstrated an increased overall survival and relapse-free survival in AML patients treated with plerixafor in combination with chemotherapy. A similar strategy was adopted in combination with hypomethylating agents such as decitabine or azacytidine^32^ or in combination with targeted drugs such as sorafenib.^33^ Treatment with sorafenib, G-CSF, and plerixafor significantly increases the mobilization of blasts and CD34^+^/CD38^−^ stem/progenitor cells, and the overall response rate as well.

## THE ROLE OF MSCs AND THEIR PROGENY IN THE REGULATION OF NORMAL HEMATOPOIESIS

In the last years, our understanding of HSCs and their niche has increased exponentially, thanks to technological advancements, including the use of reporter mice, refinement of HSC markers, single-cell RNA sequencing (scRNA-seq), CyTOF, full bone sections, and live animal imaging.^34^ Within the BM niche, HSCs are found predominantly located in perivascular regions, including sinusoidal and arteriolar vessels.^35,36^ Only a minor fraction of HSCs resides near trabecular and cortical bone surfaces.^37^ However, HSCs are motile within the niche and cannot be strictly localized within a specific area.^38^ Multiple cell types, including stromal and endothelial cells, populate perivascular regions contributing to the maintenance of HSCs. The stromal fraction includes MSCs, and cells belonging to different mesodermal lineages differentiated from them, in particular OBs and adipocytes. MSCs differ between the distinct BM regions, where periarteriolar MSCs display a propensity to undergo osteogenesis, whereas sinusoidal MSCs show an enhanced adipogenic profile.^39–41^

BM MSCs colocalize closely with HSCs and regulate their homeostasis by direct interaction through the production of soluble factors, including CXCL12, angiopoietin (ANGPT), stem cell factor (SCF), osteopontin (OPN), and interleukin 7. Moreover, MSCs protect HSCs from stress signals, such as oxidative stress and inflammation.^42^ Multiple MSC subsets with distinct impacts on HSC behavior have been identified both in mice and in human.^43^

Osteolineage cells, including pre-OBs and OBs, were among the first components of the BM niche to be associated with hematopoiesis regulation, yet the entire mechanism is still controversial. They affect HSC renewal, expansion and homing, and, depending on their stage of differentiation, their maturation along different lineages. Although the most immature uncommitted progenitor subset influences HSC maintenance and proliferation, committed progenitors or mature OBs regulate HSC differentiation along the lymphoid, myeloid, and erythroid lineages.^44^ Osteolineage cells produce many HSC-supporting molecules, including OPN, CXCL12, SCF, thrombopoietin, and ANGPT. However, recent evidence suggests that they are not the predominant source of these factors, because other populations are the main producers.^45–47^

The role of BM adipocytes in hematopoiesis regulation is complex and still under investigation.^48^ Adipocytes secrete fatty acids, hormones, adipokines, and cytokines, which have profound effects on the function of other neighboring cell populations in the BM microenvironment. Although previous studies identified BM adipocytes as negative regulators of hematopoiesis,^49^ recent works have revealed that they positively regulate HSC regeneration under stress conditions.^50^

BM endothelial cells, lining the surface of blood vessels, control vascular integrity, which in turn affects HSC trafficking and functions.^51^ The permeability of blood vessels regulates reactive oxygen species (ROS) in adjacent HSCs and niche populations, and less permeable arterioles maintain HSCs in a quiescent state with low ROS levels, whereas leaky sinusoids increase ROS levels in HSCs, thus leading to their activation and mobilization into the circulation. Endothelial cells provide soluble factors that promote HSC self-renewal and hematopoietic regeneration after injury.

The sympathetic nervous system, which innervates both the bone and the BM, also regulates HSC functions.^52^ Moreover, terminally differentiated hematopoietic cells, including megakaryocytes, macrophages, neutrophils, and regulatory T cells, play a key role in the BM niche.^44^

## BM MICROENVIRONMENT ALTERATIONS OR HSC MUTATIONS: A CHICKEN AND EGG SITUATION

AML is characterized not only by the blocking of HSC differentiation and the accumulation of blasts but also by alterations in the nonhematopoietic compartment of the BM microenvironment, which sustain leukemogenesis while suppressing normal hematopoiesis. MSCs and their progeny can contribute to leukemogenesis at least in 2 different ways: they can acquire mutations or functional alterations, caused by aging and chronic inflammation, that predispose for malignancy development, or they can be corrupted by transformed hematopoietic cells, facilitating disease manifestation and/or progression. Both these contributions can coexist during leukemia initiation and expansion.

### The transformed niche as a predisposing factor for myeloid diseases: Evidence from genetic animal models

**Figure 1.**
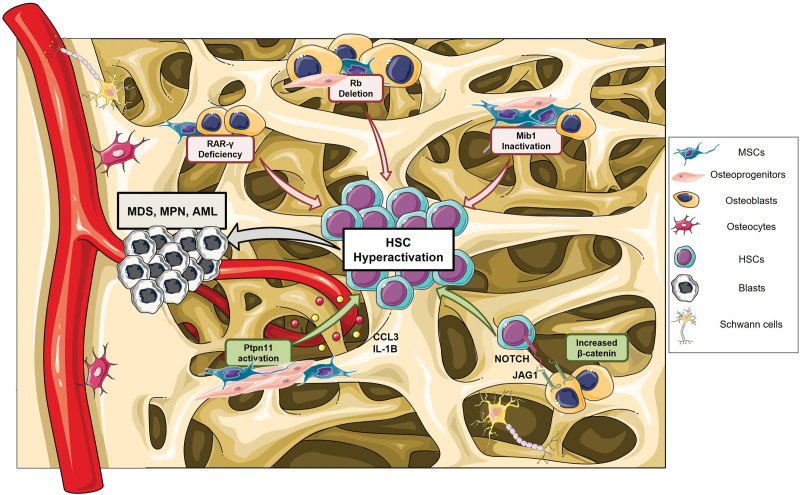
**BM stromal cell alterations that induce myeloid malignancies.** Schematic representation of altered mechanisms or mutated genes in BM stromal niche components linked to the initiation of myeloid malignancies, such as MDS, MPN, or AML in mice. Deletion of *Rb*, deficiency of *RAR*-γ, and inactivation of Mind bomb 1 (*Mib1*) genes in the stromal compartment led to MPN development. Similarly, activating mutation in tyrosine phosphatase SHP2 protein (encoded by *Ptpn11*) in osteoprogenitors increases the release of CCL3 and IL-1β hyperactivating HSCs and gives rise to MPN. Moreover, activation of β-catenin in OBs causes Jagged1 upregulation on their surface, inducing Notch1 hyperactivation in HSCs and AML onset. A mutation of *Dicer1* in osteoprogenitors leads to MDS. The figure was partially generated using Servier Medical Art, provided by Servier, licensed under a Creative Commons Attribution 3.0 unported license. AML = acute myeloid leukemia; BM = bone marrow; CCL3 = chemokine ligand 3; HSC = hematopoietic stem cell; IL = interleukin; MDS = myelodisplastic syndromes; MPN = myeloproliferative neoplasms; OB = osteoblast; RAR-γ = retinoic acid receptor-γ; Rb = retinoblastoma.

Pioneering studies on reciprocal BM transplantation using mouse models carrying mutations in nonhematopoietic or hematopoietic cells demonstrated the potential of dysregulated stroma to drive the initiation of myeloid diseases, including MPN, MDS, and AML (Figure 1). For instance, mice with retinoic acid receptor-γ-deficient (*RAR-γ*) microenvironment transplanted with wild-type (WT) BM rapidly developed MPN.^53^ Similarly, the inactivation of the Retinoblastoma (*Rb*) gene, a central regulator of the cell cycle, in both HSCs and BM microenvironment established a myeloproliferative disorder.^54^ MPN was also developed after transplanting normal HSCs into mice carrying inactivation of Mind bomb 1 (*Mib1*), the primary regulator of endocytosis of Notch ligands, in BM stromal cells.^55^

The first evidence that alterations of a specific BM stromal population represented by osteolineage cells can drive myeloid malignancies arose from the deletion of *Dicer1*, an RNAse III endonuclease, in osterix-expressing osteoprogenitors, which caused MDS-like disease with increased propensity to develop AML.^56^ *Dicer1* depletion in mature OCN-expressing OBs did not result in a hematopoietic phenotype.

Mice presenting activating mutations in the *Ptpn11* gene, encoding for the protein tyrosine phosphatase SHP2, in MSCs and osteoprogenitors but not in mature osteolineage cells showed the MPN development. These mice are characterized by the generation of a proinflammatory environment. Specifically, overproduction of the chemokine CCL3 by *Ptpn11*-mutated stromal cells can cause the recruitment of monocytes, which secreted inflammatory cytokines such as IL-1β and hyperactivated HSCs, driving the disease progression.^57^

Furthermore, constitutive activation of β-catenin in OBs leads to AML development in mice.^58^ The increased nuclear β-catenin interacts with Forkhead box protein O1 (FoxO1), inducing the activation of Notch signaling and consequently increasing the expression of the ligand Jagged1 on OBs.^59^ The subsequent aberrant activation of Notch signaling in HSCs induces their leukemic transformation and ultimately leads to AML development. Finally, it has been demonstrated that HSCs from β-catenin mutated mice acquire intrinsic alterations and, once they are transplanted in WT mice, they provoke AML onset.^58^

More recent studies provided evidence that nongenetic changes, such as BM niche remodeling caused by premature or physiologic aging, can also foster myeloid cell expansion.^60,61^ Genetic depletion of healthy mature OBs in an AML murine model causes an increase in circulating blasts and in tumor engraftment in the BM and spleen, an increase of myelopoiesis associated with a compromised B lymphopoiesis and erythropoiesis, and shortened survival.^27^ On the contrary, maintenance of the OB pool treating mice with an inhibitor of the duodenal serotonin synthesis increased mice survival, reducing circulating blasts and restoring the normal marrow function.^27^ Similar results were obtained by Bowers and colleagues^62^, which demonstrated that OB ablation significantly accelerates leukemia development and reduces the survival of transgenic BCR-ABL mice.

Osteolineage cells are not the only BM cell type involved in leukemogenesis. Loss of canonical Notch signaling in endothelial cells leads to MPN-like disease, through constitutive activation of mir-155 and NFKB signaling.^63^ Deletion of signal-induced proliferation-associated gene 1 (*Sipa1*) in mesenchymal and endothelial cells also results in MPN.^64^

Despite all these studies demonstrating that BM populations, particularly osteolineage cells, can lead to a preleukemic myeloid disease when mutated or dysfunctional in mice, similar evidence is still missing in patients.

### AML remodels the BM microenvironment favoring the generation of a self-reinforcing leukemic niche

#### Alterations in stromal cells isolated from AML patients

A possibility to approach the modifications of the human BM microenvironment in AML is to isolate and study the MSC populations derived from AML patients (AML-MSCs). Leukemic cells reprogram MSCs through cell-to-cell contact, secreted factors, and exosomes.^65–67^ Although the phenotypic profile of cell surface markers defining stromal MSCs in the BM niche does not change between healthy subjects and myeloid malignancy patients, specific subpopulation proportions, functions, or molecular profiles appear to be altered. Cytogenetic abnormalities, such as hypodiploidy, chromosomal translocations, duplications, and deletions, have been observed in AML-MSCs. Interestingly, these mutations are different from those detected in the leukemic blasts of the same individual.^68^ These alterations have also been confirmed by Kim and colleagues^69^, who described the presence of genomic instability and a global hypomethylation in AML-MSCs compared with the healthy ones. The functional consequences of these MSC alterations are still debated. Several studies carried out on MSCs from patients with AML have noted normal proliferative capacity, colony-forming unit-fibroblast capability, survival, and ability to support ex vivo hematopoiesis. In contrast, other studies showed defective hematopoietic supportive capacity, reduced expression of adhesion molecules (VCAM1 and CD49F/ITGA6), increased apoptosis and senescence, and augmented production of inflammatory cytokines in MSCs derived from patients with myeloid neoplasm.^26,65,67,69,70^ In stromal cells from MDS and AML patients, the expression of molecules involved in the interaction with HSCs is decreased, whereas the population of CD271^+^ MSCs, which favors blast expansion through upregulation of CXCL12, is increased.^26^ MSCs sustain the survival and chemoresistance of AML blasts in several ways, such as supplying prosurvival factors, providing metabolic substrates alternative to glucose, and rewiring their metabolism.^71–73^

Recent bioengineering advances were made in recreating BM stroma through organ-on-a-chip devices that would allow the investigation of MSC-mediated chemoresistance mechanisms. Biomimetic scaffolds capable of mimicking bone extracellular matrix were used to study the effects of MDS/AML blasts on MSCs, reprogramming their transcriptome toward novel prooncogenic activities.^65,67^

The capacity of MSCs derived from patients with AML to differentiate into osteolineage cells is controversial. Several studies demonstrated that AML-MSCs present an impairment in the differentiation capability into the three mesodermal lineages, and in particular in the osteogenic differentiation.^26,70,74–76^ Battula and colleagues^77^ showed that AML-MSCs present increased expression of tissue nonspecific alkaline phosphatase and a switch from adipogenic to osteogenic differentiation, driven by a BMP-dependent mechanism. Similarly, AML cells upregulated the expression of connective tissue growth factor in normal MSCs and activated Smad1/5 signaling, inducing MSCs to differentiate into committed osteoprogenitors, but not mature OBs. Our group recently demonstrated that AML-MSCs, even when removed from their pathological environment, retain an intrinsically abnormal differentiation pattern with altered osteogenesis. Using an in vivo system specific to assess their osteogenic potential, AML-MSCs exhibit a reduced capacity to form mature bone and develop an osteoprogenitor-rich niche with the presence of Osterix^+^/OCN^−^ pre-OBs and OCN^+^/Dentin matrix acid phosphoprotein 1^−^ immature osteocytes.^78^

These data indicate that AML cells educated MSCs to overproduce functionally altered osteoprogenitors, although the reasons for this differentiation impairment remain to be elucidated.

#### Evidence of AML-induced stromal niche reprogramming from animal models

**Figure 2.**
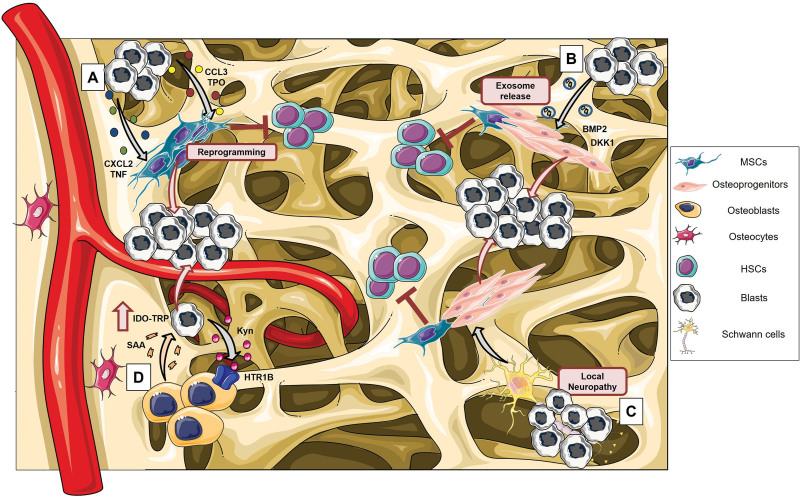
**AML-induced alterations of the BM stromal niche.** Schematic representation of mechanisms by which AML cells reprogram MSCs and their osteoblastic cell derivatives, promoting the formation of a proleukemic niche. (A) AML cells secrete soluble factors, such as TNF, CXCL2, TPO, and CCL3, which alter osteoblast formation from MSCs, impairing their HSC-supporting functions and favoring the proliferation of blasts. (B) Blasts release exosomes that are taken by mesenchymal stromal progenitors blocking their osteolineage development, reducing the support of normal hematopoiesis, and accelerating AML progression. (C) AML cells induce sympathetic neuropathy responsible for the reduction of HSC-maintaining NG2^+^ periarteriolar niche cells and the expansion of preosteoblasts, which contribute to disease progression. (D) Leukemic cells secrete oncometabolites as Kyn able to activate a self-reinforcing axis between AML cells and osteoblasts. Kyn, by binding to the serotonin receptor 1B (HTR1B), induces a proinflammatory state in osteoblasts that, through the acute-phase protein SAA, acts in a positive feedback loop on leukemia cells by increasing the expression of IDO1, enabling AML progression. The figure was partially generated using Servier Medical Art, provided by Servier, licensed under a Creative Commons Attribution 3.0 unported license. AML = acute myeloid leukemia; BM = bone marrow; CCL3 = chemokine ligand 3; CXCL2 = chemokine ligand 2; HSC = hematopoietic stem cell; IDO1 = indoleamine 2,3-dioxygenase; Kyn = kynurenine; MSCs = mesenchymal stromal cells; SAA = serum amyloid A; TNF = tumor necrosis factor; TPO = thrombopoietin.

The BM niche becomes dramatically altered during AML progression, as clearly demonstrated in many murine models of leukemia (Figure 2). The endosteal vascular niche is severely remodeled through the secretion by blasts of antiangiogenic and inflammatory cytokines, such as tumor necrosis factor and chemokine ligand 2 (CXCL2), which impair stromal cells, endothelial cells, and OBs, reducing their capacity to sustain normal HSC maintenance.^79^ The ability of AML cells to alter BM vasculature is further supported by the extensive vascular remodeling observed in PDX models and BM biopsies from patients.^25,28^ Interestingly, the rescue of endosteal vessels preserves HSCs and improves chemotherapy efficacy.^79^ In addition, Xiao et al demonstrated that AML MLL-AF9 cell infiltration in the BM causes an expansion of Early B-cell factor 2^+^ (Ebf2^+^) MSCs with reduced CXCL12 expression, which favors AML establishment.^80^

Furthermore, leukemic myeloid cells alter the fine control of the osteogenic niche,^81,82^ increasing the number of functionally altered osteoprogenitor cells and immature OBs. Blocking the terminal differentiation of MSCs into mature OBs seems to contribute to AML progression. In an MLL-AF9 AML model, the leukemia development causes local neuropathy, reducing the number of NG2^+^ periarteriolar cells, which are involved in the maintenance of HSC quiescence, and leading to the osteogenic differentiation of Nestin^+^ MSCs. Despite this commitment to osteoblastic lineage, these mice show a lack of terminally differentiated OBs and deficient bone mineralization.^83^ Similar results were reported also for a JAK2V617F-MPN mouse model, in which sympathetic neuropathy causes a reduction of Nestin^+^ MSCs, which promotes the expansion of mutant HSCs and facilitates disease progression by loss of HSC-retention factor expression, including CXCL12, SCF, ANGPT1, and VCAM1.^84^ Frisch and colleagues^81^, using an immunocompetent murine model of leukemia, demonstrated that AML blasts alter directly normal OB functions. Engrafted animals presented decreased OPN-positive OBs, loss of trabecular structures, and lower serum levels of the bone formation marker OCN. Osteoblastic inhibition by leukemia seems to be mediated by chemokine CCL3, which is increased in malignant BM cells, both in leukemic mice and patients.^81^ Furthermore, FLT3-ITD-positive mice showed alterations in bone morphology suggesting a possible impact of aberrant oncogenic FLT3 signaling on the process controlling bone homeostasis.^85,86^

Kumar and colleagues^66^ noted that AML patients present an increase in exosome secretion, which correlates with the reduction of OCN plasma levels. Moreover, transplantation of AML-derived exosomes induces in the murine BM microenvironment alterations similar to those described for mice engrafted with AML cells (eg, block of osteolineage maturation and reduced bone formation). Interestingly, AML-derived exosomes transfer to the BM stroma the negative regulator of osteogenesis Dickkopf-1 (DKK1), which causes suppression of OB differentiation and supports a more rapid leukemia progression. Treatment of transplanted mice with DKK1 inhibitors significantly delays AML progression and increases their overall survival. In addition, AML-derived exosomes induce downregulation of HSC-supporting factors (eg, CXCL12, KITL, and IGF1) in BM stromal cells, reducing their capacity to support normal hematopoiesis.^66^ Doron and colleagues^87^ demonstrated that AML cells can use extracellular vesicles to transmit endoplasmic reticulum stress and to promote the unfolded protein response in stromal cells, inducing their osteogenic commitment partially through the transfer of BMP2.

Recently, Galan-Diez and colleagues^88^ identified kynurenine (Kyn), a tryptophan catabolite, as a new promoter of the BM niche remodeling in AML. AML-secreted Kyn binds to the serotonin receptor 1B (HTR1B) on OBs, inducing a proinflammatory state characterized by increased expression of acute-phase protein serum amyloid A (SAA). SAA acts with a mechanism of positive feedback on leukemia cells by increasing the expression of indoleamine 2,3-dioxygenase-1 (IDO1), the rate-limiting enzyme for Kyn synthesis, thereby enabling AML progression. Genetic and pharmacological inhibition of kyneurin-HTR1B interaction between leukemia cells and OBs hampers AML proliferation.

As the study of the BM niche contribution to leukemia development is hindered in humans, reliable models of the human leukemic niche are fundamental for the confirmation of these findings and their translational application. In this regard, humanized BM niche models offer a valuable alternative. Over the years, several models using different scaffold materials and implant techniques have been proposed to recreate a humanized BM microenvironment that allows studying the ability of human stromal cells to sustain the normal and/or leukemic engraftment and to evaluate the interactions between hematopoietic and nonhematopoietic compartments.^65,67,89,90^ Battula and colleagues^77^ developed a human AML niche model, which consists of subcutaneous implantation of human bone fragments freshly collected from healthy subjects into the flanks of NSG mice transplanted with human AML cell lines. The authors demonstrated that mice transplanted with human AML cell lines presented a strong upregulation of early-stage OB markers *Runx2* and *Osterix* in both the endosteal surface and medullary cavity of implants, compared with those harvested from control mice, confirming an increased but incomplete osteogenic activity also in humanized leukemic BM.

## UNRAVELING THE COMPLEXITY OF THE LEUKEMIC NICHE BY OMICS

Comprehensive mapping of the interactions occurring within the BM niche during AML pathogenesis and treatment would have a paramount impact on developing new therapeutic approaches targeting the leukemic microenvironment, aimed at improving the patient outcome. Although the impact of AML on transcriptomic changes in the immune microenvironment has recently been addressed in patients,^91^ very little is known about the nonhematopoietic niche in human BM at the molecular level.

scRNA-seq^39,40,92^ and spatially resolved transcriptomics^41^ have recently allowed to unravel the complexity of heterogeneous stromal progenitors in murine BM. Under homeostatic conditions, all these studies found considerable overlap between the identified niche populations. To examine how nonhematopoietic BM cells are altered during AML development, Baryawno and colleagues^39^ compared scRNA-seq profiles from healthy and MLL-AF9-knock-in leukemic mice. They identified 17 distinct BM populations at steady state, including 3 endothelial (sinusoids, arteries, and arterioles), 1 pericyte, 2 osteolineage (OLC-1 and OLC-2), 5 fibroblastic, 1 mesenchymal (LepR-MSCs), and 4 chondrocyte clusters. The MLL-AF9 mouse model showed significant alterations in the cellular composition of the BM microenvironment, with a marked decrease in osteolineage-differentiated LepR-MSCs and a concurrent increase in pre-OBs. This suggests an impairment in osteogenic differentiation due to the induction of genes inhibiting bone formation and calcification and is consistent with the decrease of bone formation and OB number reported in murine models of AML.^66,83^ Even the adipogenic differentiation of LepR-MSCs was impaired as revealed by the downregulation of several adipocytic-related genes. This is in line with a previous study reporting defective adipogenesis in AML PDX models.^93^ In addition, the presence of leukemic cells reduced the expression of regulatory molecules necessary for normal hematopoiesis in specific stromal subsets, including endothelial cells.

With a similar approach, Passaro and colleagues^92^ examined the changes affecting BM stromal cells during AML development using an AML PDX model.^92^ They showed that major niche components, including CD31^+^ cells, Col1a1^+^ cells, and Nestin^+^ cells, which overlapped with the signature of endothelial, skeletal, and smooth muscle cells, respectively, underwent gene expression changes upon AML engraftment. Integrating BM transcriptome and secretome, they described a deregulated proteomic signature similar to the one reported for AML patients.^94^ Interestingly, several signaling mediators of the complement and coagulation cascade result downregulated, in agreement with clinical evidence of bleeding disorders and prothrombotic state in AML patients. Moreover, mediators associated with neutrophil degranulation are reduced, while chemokines involved in monocyte chemotaxis results increased, suggesting alterations in the regulation of innate immunity.

Similarly, a scRNA-seq study on BM stroma of mice with MPN reported a functional reprogramming of 2 specific MSC subsets into collagen-producing myofibroblasts, responsible for extracellular matrix deposition and fibrosis. Inflammation plays a critical role, as increased expression of alarmin heterodimer S100A8/S100A9 is related to stroma reprogramming from hematopoiesis support toward fibrotic transformation.^95^

Very recently, a scRNA-seq approach has been applied to deconvolute the heterogeneity of MSCs in healthy human BM, leading to similar findings compared with the murine experiments, revealing adipo-, osteo-, and chondrogenic clusters.^96,97^ Similarly, the analysis of the stromal compartment in BM of multiple myeloma patients led to the identification of a myeloma-specific inflammatory MSC subset, which spatially colocalized with tumor and immune cells and showed the expression of genes involved in tumor survival and immune modulation.^98^ These studies represent the first steps to unraveling alterations of BM stroma components during the development of hematological malignancies in humans.

Although RNA-seq studies are useful, proteome-based studies would be essential to investigate molecules that are regulated by posttranscriptional mechanisms. Çelik and colleagues^94^ performed proteomic profiling of the noncellular soluble compartment of the BM microenvironment in patients with AML using the SOMAscan assay. They found 91 upregulated and 77 downregulated proteins in the leukemic BM. The proteomic signature of the AML BM niche revealed perturbation of signaling networks, particularly those associated with chemokine and cytokine signaling. Specifically, levels of proteins involved in bone homeostasis such as BMP10, CCL3, CX3CL1, OPN, endoglin, parathyroid hormone-like hormone, the secreted regulators of BMP signaling pathway, chordin-like protein 1, and matrix metalloproteinase 7 displayed altered values.

Although more effort is necessary to unravel the BM stromal cell changes in different AML stages, all transcriptomic and proteomic data indicate a shift toward an accumulation of pre-OBs that might be instrumental in disease evolution and should be further explored to identify novel therapeutic targets.

## CONCLUSION AND FUTURE PERSPECTIVES

The studies reviewed herein, largely performed in animal models, highlight that leukemic myeloid cells and the BM stroma interact extensively, having mutual effects on each other. However, despite a growing set of evidence in patients sustains that leukemic cells can alter the stromal compartment by producing factors that corrupt the BM niche to their advantage, the role of somatic mutations in BM stromal cells in leukemia initiation has not yet been confirmed in patients.

The elucidation of mechanisms behind the interaction between AML cells and BM stroma can represent a turning point in AML treatment. Indeed, despite the improvements in the management of this disease, AML patients still face a risk of chemoresistance and relapse that severely affect the outcome.^3^ The ultimate goal of research in the field of hematologic malignant niches is the development of therapies targeting the deregulated microenvironment, which, alone or in combination with the existing cell-intrinsic therapies, may increase leukemia susceptibility to treatment, leading to the complete eradication of all malignant clones. This could be accomplished by targeting the interactions between blasts and their niche, or blast-intrinsic pathways that influence the niche or directly the niche components. Because several mechanisms involved in leukemia progression often overlap across multiple leukemic subtypes, niche-oriented therapeutic targets would be effective for a wide cohort of patients. Recent evidence about the main role of pre-OBs on AML progression led to considering the crosstalk taking place between blasts and osteolineage cells as a potential target for therapy.

Our knowledge about cellular populations and molecular mechanisms of the BM niche involved in interactions with healthy and malignant hematopoiesis is still in its infancy. For this reason, the application of new imaging and sequencing technologies for the analysis of BM samples from patients represents a valuable option not only to shed light on the pathogenesis of myeloid neoplasms but also to identify possible novel therapeutic targets. Furthermore, monitoring the specific signature of the leukemic niche at diagnosis and during therapy could be exploited as a potential tool to predict response in clinical trial settings.

## ACKNOWLEDGMENTS

The authors thank Fondazione Matilde Tettamanti and Comitato Maria Letizia Verga for their generous and constant support.

## AUTHOR CONTRIBUTIONS

CT, BR, and AP wrote the article. AB and MS revised and edited the article. All authors have read and agreed to the final version of the article.

## DISCLOSURES

The authors have no conflicts of interest to disclose.

## SOURCES OF FUNDING

This work was partially supported by AIRC 5x1000 “Immunity in Cancer Spreading and Metastasis (ISM)” (grant 2018-21147) to AB, AIRC IG 2022 (grant 2022-27507) to MS, PRIN 2021-NAZ-0033 to AB, and by Associazione SKO Arianna Amore ONLUS (research fellowship to CT).
